# Emerging and Zoonotic Virus Challenges of Developing Nations

**DOI:** 10.2174/1874357901812010042

**Published:** 2018-08-31

**Authors:** Yashpal Singh Malik, Kuldeep Dhama, Raj Kumar Singh

**Affiliations:** 1 ICAR-Indian Veterinary Research Institute, Izatnagar 243122, India; 2 ICAR-Indian Veterinary Research Institute, Bareilly 243122, Uttar Pradesh, India; 3 ICAR-Indian Veterinary Research Institute, Bareilly 243122, Uttar Pradesh, India

The word “Emerging Diseases” encompasses the potential threat posed by infectious pathogens to humans and animals. In the recent times, several epidemics and few pandemics of viral origin have haunted the global population. Majorly, the transboundary and zoonotic pathogens are of concerns to the public health. Most recently, Zika virus has been found with confounding situation in Asian countries. Correspondingly, other infectious viruses like Ebola virus, SARS, MERS, Dengue, Chicken Guinea, West Nile and Japanese encephalitis have created panic owing to their severe life-threatening consequences, affecting larger population globally with a high toll of human deaths, gaining potential of rapid spread across borders of many countries, and creating economic impacts and high sufferings to the mankind. Of note, nearly two-thirds of these viral pathogens have been of zoonotic nature having significant public health concerns. Predisposing factors such as deforestation, urbanization, ecotourism, fast population movement, changing climatic issues (global warming), and ecological alterations with the evolution of newer viral pathogens /strains and jumping the host species barriers, *etc*, altogether they have increased the human and animals interface including wildlife, which has lured up a number of infectious viral diseases. Generally, viral pathogens remain under-diagnosed under the limited health services, lack of awareness and poorly coordinated health efforts, especially in the developing and under-developed countries. The research advances made during the last two decades are paving novel and effective ways to tackle such infectious agents in a better way. Additionally, enhanced disease surveillance and monitoring, rapid diagnostics, inventing effective prophylactics, and adapting appropriate public health measures would certainly help in combating the emerging viral threats.

In this special issue on “Emerging and Zoonotic Virus Challenges of Developing Nations”, a review article by N. Kobayashi from Japan has highlighted various factors which may synergistically increase risks for the emergence of pathogens, transmission of pathogens, and opportunity of infections. Added to this, he pointed out that the spread of emerging viruses might be indirectly related to socioeconomic problems including civil wars, increase of refugees, and natural disasters. These human factors always compromise the human health and increase the risk for emerging infectious diseases. Despite such situations, scientists have the significant role to reduce the risks for the emerging viral diseases. This article emphasizes the first priority to ensure adequate surveillance of viral diseases, *i.e*., to maintain epidemiological study on humans, animals, virus strains from various sources including environment, which is relevant to “One Health” approach advocated recently. In the recent years, Chandipura Virus (CHPV) has emerged as an encephalitic pathogen and found associated with a number of outbreaks in different parts of India. The article by Sapkal *et al*. narrate the overview on CHPV focusing on prevalence and seasonal activity of this virus in India through outbreak investigations, serosurvey, and diagnosis of the referred clinical specimens.

Several semi-captive/free-ranging wild avian species have been noted playing a vital role in the dissemination of viruses, which is an important consideration to control the disease outbreaks. In this series, Newcastle disease (ND), caused by Avian Paramyxovirus serotype 1 (APMV-1), is a notifiable disease throughout the world due to the economic impact on trading restrictions and its embargoes placed in endemic regions. The article by Rahman *et al*. highlights the adaptation of Newcastle Disease Virus (NDV) in feral birds and their potential role in interspecies transmission. The review details the pertinent features of several historical and contemporary aspects of NDV and the vital role of feral birds in its molecular epidemiology and ecology. Reports are very less on the existence of Bovine Viral Diarrhea Virus (BVDV) in pigs. The North Eastern (NE) part of India is having the maximum pig population in India and the article by Chakraborty *et al*. emphasized on the BVDV in pigs from this region of India with genetic profiling of virus strains. 

One of the viral diseases of zoonotic concern, Japanese Encephalitis (JE) has recently been declared as a notifiable disease in India due to its expanding geographical distribution, on which, the article by Kulkarni *et al*. has given a comprehensive Indian perspectives over highlighting the recent progress, focusing challenges with diagnosis and prophylactic interventions**. **Lately, Antimicrobial Resistance (AMR) or Multidrug Resistance (MDR) has become a serious health issue, globally. Even resitance in virus has been noted. The review article on “The Quest for Materials-based Hydrogels with Antimicrobial and Antiviral Potentialities” by Iqbal highlights the current concepts in this area. The review has pin-pointed various methodological approaches including biomaterials-based therapeutic hydrogels aiming to resolve these issues. The information is also given on the potential research activities, and possible mechanisms of actions of hydrogels are discussed with a closeup look at the future recommendations. In recent years, the two porcine viral diseases have gained the attraction of researchers due to the losses associated with them and influence of concurrent infections. In this area, the research group of Mukherjee *et al*. investigated the expression of various cytokines including IL2, INF_G, IL-10, TNF_Beta, IL12 and TNF Alpha by quantitative reverse transcription-polymerase chain reaction in growing piglets based on the viral load of two main porcine viruses namely porcine circovirus 2 and classical swine fever virus to define the influence of co-infection on host immunity. Another article on “Zoonotic viral diseases of equines” by Kumar *et al*. provided an overview of the infectious zoonotic diseases and their impact on human and animal health. Among the emerging viral infections, a new addition is the Picobirnavirus, which has been documented in enteric and respiratory infections in several mammalian and avian host species. It is an opportunistic infection seen more commonly with immune-compromised individual with concurrent infection. The review article by Malik *et al*. emphasized on its relevance to Indian human and animal population. This viral infection of wide host range needs the attention of researchers globally to find out its control measures. 

This thematic issue will serve as a scientific platform to share the knowledge on emerging infectious viral diseases which are showing added prominence in the 21^st^ century. The compilation of articles included in this issue would be helpful for the public health professionals, clinicians, animal owners, epidemiologists, academicians, researchers and policymakers in formulating the strategies for adopting appropriate preventive and control measures for the prevailing and upcoming viral diseases.

